# (*E*)-*N*-(4-Bromo­phen­yl)-1-(quinolin-2-yl)methanimine

**DOI:** 10.1107/S2414314626008953

**Published:** 2026-09-03

**Authors:** Sizwe J. Zamisa, Adesola A. Adeleke, Bernard Omondi, Olatunde S. Olatunji

**Affiliations:** a School of Agriculture and Science, Discipline of Chemistry, University of KwaZulu-Natal, Private Bag X54001, Durban, 4000, South Africa; b Department of Chemical Sciences, Olabisi Onabanjo University, Ago-Iwoye, PMB 2001, Nigeria; Goethe-Universität Frankfurt, Germany

**Keywords:** crystal structure, Schiff base, quinoline, imine, 4-bromo­phen­yl

## Abstract

The title compound, C_16_H_11_BrN_2_, is a Schiff base formed through the condensation reaction of 2-quinoline­carboxaldehyde with 4-bromo­aniline. The C=N bond length of 1.282 (2) Å is consistent with its double-bond character. The imine linkage adopts the *E* configuration, as indicated by the N—C—C—N torsion angle of 174.27 (14)°. This value also demonstrates that the quinoline ring and imine group are arranged in an almost coplanar manner. In contrast, the 4-bromo­phenyl ring is significantly twisted relative to the imine-quinoline plane, as indicated by the C—N—C—C torsion angle of approximately 46.8 (2)°. Inter­molecular C—H⋯π inter­actions between quinolinyl and bromo­phenyl rings form a three-dimensional supra­molecular network in the crystal packing.

## Abstract 2

The title Schiff base was prepared by condensation of 2-quinoline­carboxaldehyde with 4-bromo­aniline, is reported. Inter­molecular C—H⋯π inter­actions between quinolinyl and bromo­phenyl rings form a three-dimensional supra­molecular network in the crystal packing.

## Graphical abstract

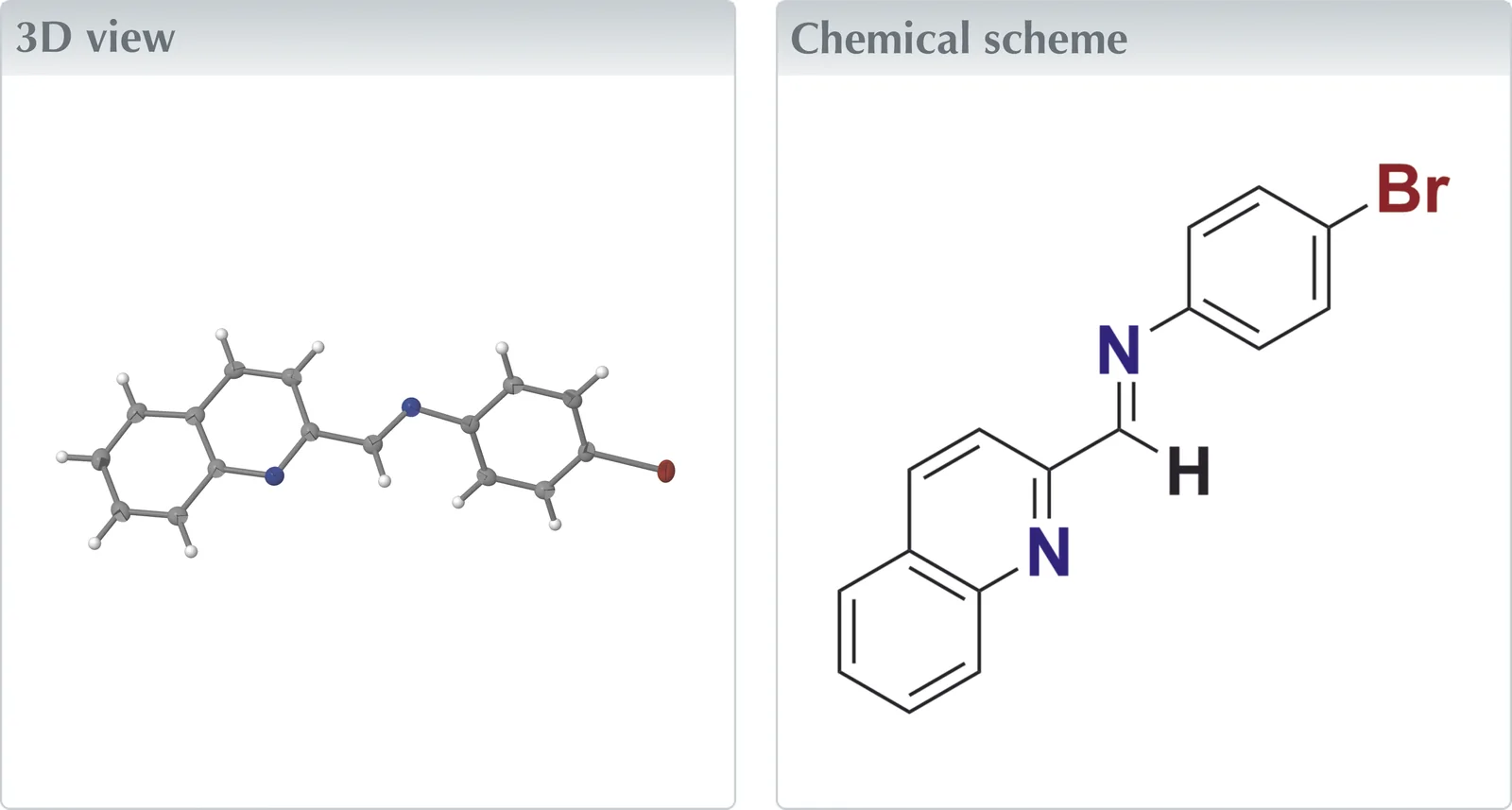

## Structure description

Schiff base compounds derived from aromatic aldehydes and primary amines constitute an important class of organic ligands that have been extensively investigated owing to their ease of preparation, structural versatility, and broad biological and pharmacological profiles, including anti­bacterial, anti­fungal, and anti­cancer activities (Boulechfar *et al.*, 2023 ▸; Adeleke *et al.*, 2022 ▸). Among the various aldehyde building blocks employed, quinoline­carboxaldehyde is of particular inter­est because it incorporates the quinoline pharmacophore, a privileged scaffold in medicinal chemistry known for its activity against a wide range of pathogens and tumour cell lines (Solomon *et al.*, 2011 ▸). The resulting quinoline-derived Schiff bases combine the chelating and biological potential of the quinoline unit with the donor flexibility of the imine nitro­gen, rendering them attractive precursors for metal-complex synthesis and functional material applications (Sonawane *et al.*, 2023 ▸).

The asymmetric unit of the title compound consists of a single, neutral mol­ecule of (*E*)-*N*-(4-bromo­phen­yl)-1-(quinolin-2-yl)methanimine. The mol­ecule comprises a quinoline ring system connected at its 2-position to a methanimine (–CH=N–) bridge, which in turn links to a 4-bromo­phenyl ring at the imine nitro­gen (Fig. 1 ▸). The quinoline ring system and the imine fragment are coplanar, as the torsion angles within the quinoline ring confirm near-perfect planarity of this moiety, and the imine carbon lies in this plane, as reflected by the torsion of −2.9 (2)°. In contrast, the 4-bromo­phenyl ring is significantly twisted with respect to the quinoline–imine plane, as shown by the torsion angle C10—N2—C11—C12 of 46.8 (2)°. Other intra­molecular bond parameters are comparable to closely related quinoline-based Schiff bases reported in the literature (Oladipo *et al.*, 2025 ▸; Wang *et al.*. 2017*a* ▸,*b* ▸; Faizi *et al.*, 2015 ▸). The crystal packing of the title compound features C–H⋯π inter­actions between the quinolinyl and bromo­phenyl rings of neighbouring mol­ecules, forming a three-dimensional supra­molecular network. (Table 1 ▸, Fig. 2 ▸).

## Synthesis and crystallization

The title compound was synthesized by the condensation reaction of an equimolar amount of 2-quinoline­carboxaldehyde with 4-bromo­aniline in methanol, with a catalytic amount of glacial acetic acid. The reaction mixture was refluxed for 6 h at 80°C for 4 h, during which time a yellow precipitate was formed. The solid product was isolated by filtration, washed with cold ethanol, and dried in air (Adeleke *et al.*, 2021 ▸). Single crystals were obtained by slow evaporation of the concentrated solution of the product in ethanol at room temperature.

## Refinement

Crystal data, data collection and structure refinement details are summarized in Table 2 ▸.

## Supplementary Material

Crystal structure: contains datablock(s) I. DOI: 10.1107/S2414314626008953/bt4206sup1.cif

Structure factors: contains datablock(s) I. DOI: 10.1107/S2414314626008953/bt4206Isup2.hkl

Supporting information file. DOI: 10.1107/S2414314626008953/bt4206Isup3.cml

CCDC reference: 2583971

Additional supporting information:  crystallographic information; 3D view; checkCIF report

## Figures and Tables

**Figure 1:**
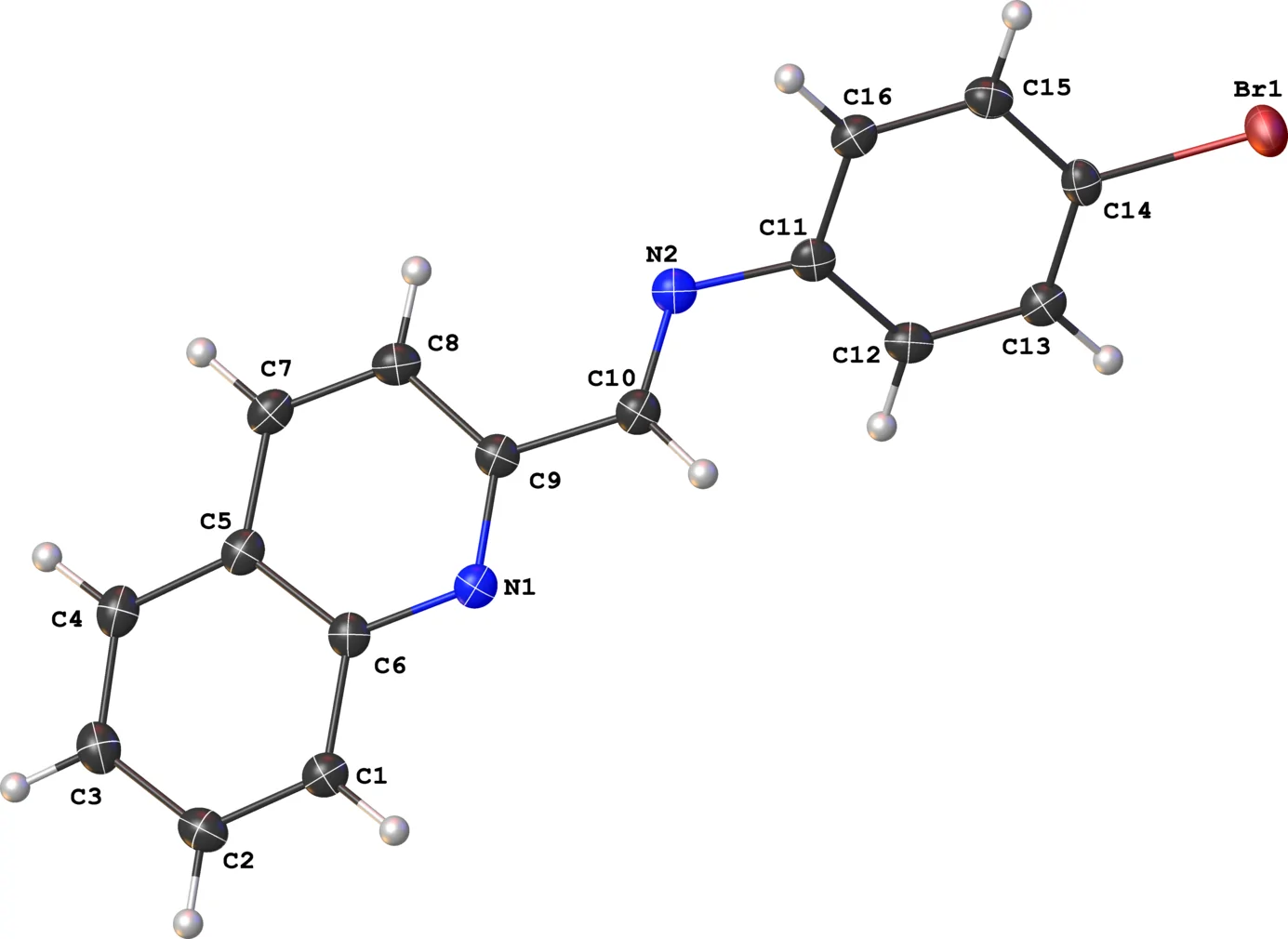
The mol­ecular structure of the title compound shown with displacement ellipsoids at the 50% probability level.

**Figure 2:**
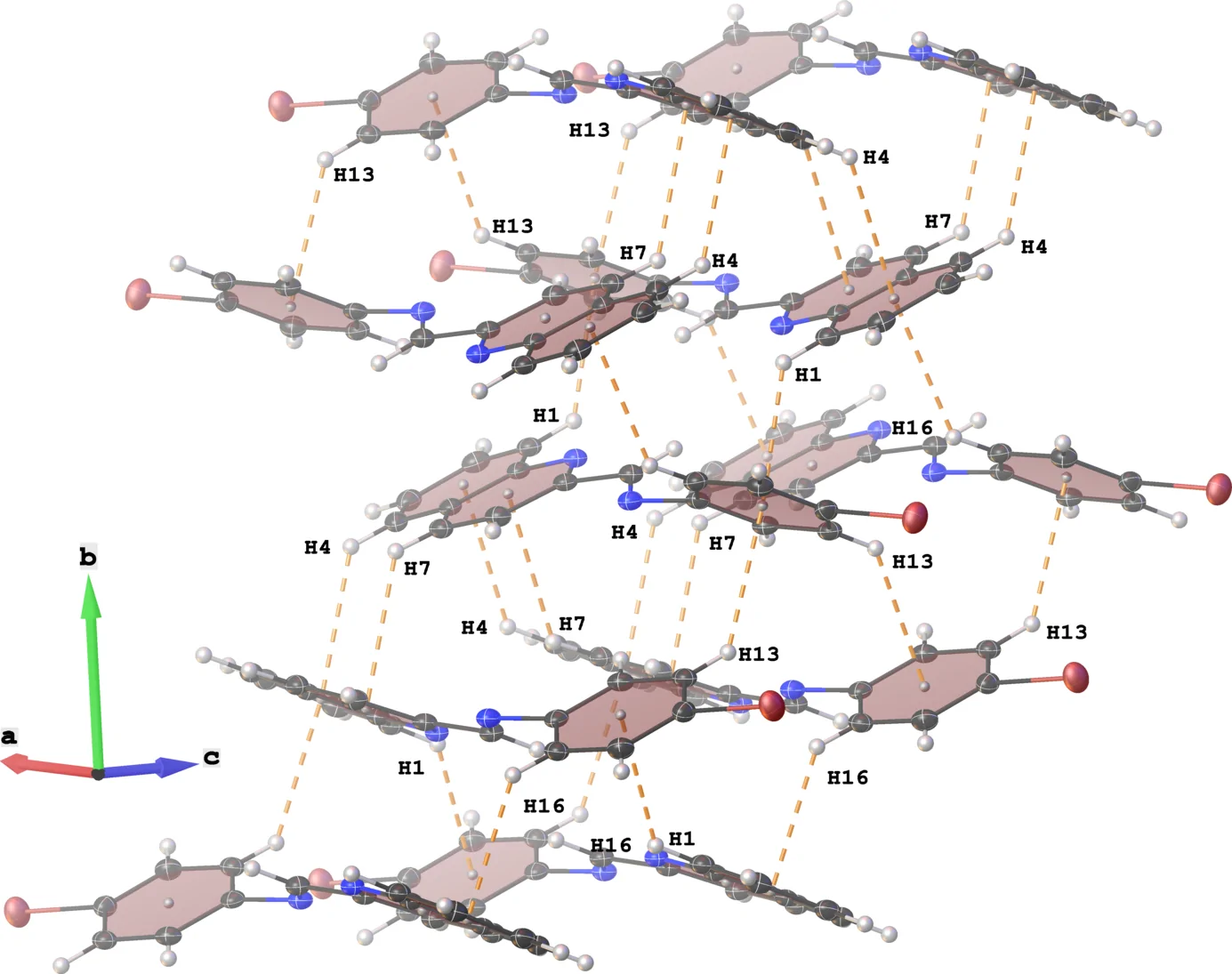
Representation of the C—H⋯π hydrogen bonds in the crystal structure of the title compound (dashed orange lines).

**Table 1:** Hydrogen-bond geometry (Å, °) *Cg*1, *Cg*2 and *Cg*3 are the centroids of the N1/C5–C9, C1–C6 and C11–C16 rings, respectively.

*D*—H⋯*A*	*D*—H	H⋯*A*	*D*⋯*A*	*D*—H⋯*A*
C1—H1⋯*Cg*3^i^	0.95	2.67	3.3703 (16)	131
C4—H4⋯*Cg*2^ii^	0.95	2.79	3.4808 (17)	130
C7—H7⋯*Cg*1^ii^	0.95	2.85	3.5026 (16)	127
C13—H13⋯*Cg*3^iii^	0.95	2.69	3.4042 (16)	132
C16—H16⋯*Cg*2^iv^	0.95	2.70	3.3932 (15)	131

Symmetry codes: (i) 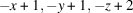; (ii) 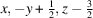; (iii) 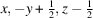; (iv) 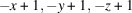.

**Table 2:** Experimental details

Crystal data	
Chemical formula	C_16_H_11_BrN_2_
*M*_r_	311.18
Crystal system, space group	Monoclinic, *P*2_1_/*c*
Temperature (K)	100
*a*, *b*, *c* (Å)	15.3817 (4), 13.9338 (4), 6.0050 (2)
β (°)	91.654 (1)
*V* (Å^3^)	1286.49 (7)
*Z*	4
Radiation type	Cu *K*α
μ (mm^−1^)	4.23
Crystal size (mm)	0.42 × 0.33 × 0.05

Data collection	
Diffractometer	Bruker APEXII CCD
Absorption correction	Multi-scan (*SADABS*; Krause *et al.*, 2015 ▸)
*T*_min_, *T*_max_	0.545, 0.753
No. of measured, independent and observed [*I* > 2σ(*I*)] reflections	19912, 2346, 2224
*R*_int_	0.040
(sin θ/λ)_max_ (Å^−1^)	0.602

Refinement	
*R*[*F*^2^ > 2σ(*F*^2^)], *wR*(*F*^2^), *S*	0.023, 0.062, 1.05
No. of reflections	2346
No. of parameters	172
H-atom treatment	H-atom parameters constrained
Δρ_max_, Δρ_min_ (e Å^−3^)	0.22, −0.45

Computer programs: *APEX2* and *SAINT* (Bruker, 2009 ▸), *SHELXT2018/2* (Sheldrick, 2015*a* ▸), *SHELXL2019/3* (Sheldrick, 2015*b* ▸) and *OLEX2* 1.5 (Dolomanov *et al.*, 2009 ▸).

## full crystallographic data

### (E)-N-(4-Bromophenyl)-1-(quinolin-2-yl)methanimine Crystal data

**Table:**

C_16_H_11_BrN_2_	*F*(000) = 624
*M**_r_* = 311.18	*D*_x_ = 1.607 Mg m^−^^3^
Monoclinic, *P*2_1_/*c*	Cu *K*α radiation, λ = 1.54178 Å
*a* = 15.3817 (4) Å	Cell parameters from 9977 reflections
*b* = 13.9338 (4) Å	θ = 2.9–68.1°
*c* = 6.0050 (2) Å	µ = 4.23 mm^−^^1^
β = 91.654 (1)°	*T* = 100 K
*V* = 1286.49 (7) Å^3^	Plate, colourless
*Z* = 4	0.42 × 0.33 × 0.05 mm

### (E)-N-(4-Bromophenyl)-1-(quinolin-2-yl)methanimine Data collection

**Table:**

Bruker APEXII CCD diffractometer	2346 independent reflections
Radiation source: microfocus sealed X-ray tube, Incoatec Iµs	2224 reflections with *I* > 2σ(*I*)
Mirror optics monochromator	*R*_int_ = 0.040
Detector resolution: 7.9 pixels mm^-1^	θ_max_ = 68.1°, θ_min_ = 2.9°
φ and ω scans	*h* = −18→17
Absorption correction: multi-scan (SADABS; Krause *et al.*, 2015) wR2(int) was 0.1018 before and 0.0553 after correction. The ratio of minimum to maximum transmission is 0.7237. The λ/2 correction factor is 0.0015.	*k* = −16→16
*T*_min_ = 0.545, *T*_max_ = 0.753	*l* = −6→7
19912 measured reflections	

### (E)-N-(4-Bromophenyl)-1-(quinolin-2-yl)methanimine Refinement

**Table:**

Refinement on *F*^2^	0 restraints
Least-squares matrix: full	Hydrogen site location: inferred from neighbouring sites
*R*[*F*^2^ > 2σ(*F*^2^)] = 0.023	H-atom parameters constrained
*wR*(*F*^2^) = 0.062	*w* = 1/[σ^2^(*F*_o_^2^) + (0.0377*P*)^2^ + 0.4867*P*] where *P* = (*F*_o_^2^ + 2*F*_c_^2^)/3
*S* = 1.05	(Δ/σ)_max_ = 0.001
2346 reflections	Δρ_max_ = 0.22 e Å^−^^3^
172 parameters	Δρ_min_ = −0.45 e Å^−^^3^

### (E)-N-(4-Bromophenyl)-1-(quinolin-2-yl)methanimine Special details

**Table:**

Geometry. All esds (except the esd in the dihedral angle between two l.s. planes) are estimated using the full covariance matrix. The cell esds are taken into account individually in the estimation of esds in distances, angles and torsion angles; correlations between esds in cell parameters are only used when they are defined by crystal symmetry. An approximate (isotropic) treatment of cell esds is used for estimating esds involving l.s. planes.
Refinement. Hydrogen atoms were located in a difference map and refined as riding on their parent atom with *U*(H) = 1.2*U*_eq_(C) and with C—H = 0.95 Å.

### (E)-N-(4-Bromophenyl)-1-(quinolin-2-yl)methanimine Fractional atomic coordinates and isotropic or equivalent isotropic displacement parameters (Å^2^)

**Table:**

	*x*	*y*	*z*	*U*_iso_*/*U*_eq_	
Br1	0.03190 (2)	0.63813 (2)	1.15570 (3)	0.02712 (9)	
N1	0.59463 (8)	0.58283 (8)	0.7717 (2)	0.0175 (3)	
N2	0.37000 (9)	0.61932 (9)	0.6575 (2)	0.0185 (3)	
C13	0.21335 (10)	0.66459 (10)	1.1058 (3)	0.0187 (3)	
H13	0.211684	0.692263	1.250160	0.022*	
C7	0.60856 (10)	0.65857 (10)	0.3381 (3)	0.0194 (3)	
H7	0.612642	0.682644	0.190800	0.023*	
C5	0.68526 (11)	0.64033 (9)	0.4694 (3)	0.0176 (3)	
C8	0.52919 (11)	0.64130 (9)	0.4251 (3)	0.0197 (3)	
H8	0.477397	0.653464	0.339721	0.024*	
C4	0.76976 (11)	0.66208 (11)	0.3979 (3)	0.0204 (3)	
H4	0.777239	0.689339	0.254716	0.024*	
C1	0.74967 (10)	0.58019 (10)	0.8203 (3)	0.0187 (3)	
H1	0.743555	0.551406	0.962403	0.022*	
C6	0.67467 (10)	0.60071 (10)	0.6855 (2)	0.0167 (3)	
C14	0.13806 (11)	0.62969 (10)	1.0021 (3)	0.0187 (3)	
C10	0.44132 (10)	0.59306 (10)	0.7552 (2)	0.0187 (3)	
H10	0.440070	0.565713	0.900039	0.022*	
C11	0.29350 (10)	0.61891 (10)	0.7815 (3)	0.0173 (3)	
C9	0.52518 (9)	0.60476 (11)	0.6458 (2)	0.0172 (3)	
C12	0.29111 (10)	0.65845 (10)	0.9953 (3)	0.0183 (3)	
H12	0.343228	0.681321	1.065495	0.022*	
C16	0.21614 (10)	0.58570 (10)	0.6789 (2)	0.0186 (3)	
H16	0.217073	0.560089	0.532372	0.022*	
C15	0.13827 (10)	0.58990 (10)	0.7894 (3)	0.0199 (3)	
H15	0.086093	0.566078	0.721322	0.024*	
C3	0.84113 (11)	0.64401 (10)	0.5343 (3)	0.0217 (3)	
H3	0.897616	0.659973	0.486127	0.026*	
C2	0.83087 (10)	0.60168 (11)	0.7465 (3)	0.0212 (3)	
H2	0.880681	0.588095	0.838352	0.025*	

### (E)-N-(4-Bromophenyl)-1-(quinolin-2-yl)methanimine Atomic displacement parameters (Å^2^)

**Table:**

	*U*^11^	*U*^22^	*U*^33^	*U*^12^	*U*^13^	*U*^23^
Br1	0.01970 (13)	0.03373 (14)	0.02817 (13)	−0.00120 (6)	0.00494 (8)	−0.00493 (6)
N1	0.0213 (6)	0.0120 (6)	0.0193 (6)	0.0004 (5)	0.0011 (5)	0.0001 (5)
N2	0.0214 (7)	0.0127 (5)	0.0214 (6)	−0.0001 (5)	0.0009 (5)	−0.0005 (5)
C13	0.0238 (8)	0.0124 (6)	0.0196 (7)	0.0003 (6)	−0.0003 (6)	−0.0003 (6)
C7	0.0270 (8)	0.0133 (6)	0.0180 (7)	0.0010 (6)	0.0005 (6)	0.0003 (6)
C5	0.0239 (8)	0.0103 (7)	0.0187 (8)	−0.0004 (5)	0.0019 (6)	−0.0023 (5)
C8	0.0229 (8)	0.0147 (7)	0.0212 (8)	0.0002 (5)	−0.0024 (6)	0.0000 (5)
C4	0.0268 (8)	0.0136 (6)	0.0211 (8)	−0.0023 (6)	0.0053 (6)	−0.0021 (6)
C1	0.0233 (8)	0.0126 (6)	0.0203 (7)	0.0007 (5)	−0.0001 (6)	−0.0008 (5)
C6	0.0202 (7)	0.0101 (6)	0.0198 (7)	−0.0003 (5)	0.0020 (6)	−0.0017 (5)
C14	0.0184 (8)	0.0154 (7)	0.0224 (8)	0.0017 (5)	0.0037 (6)	0.0013 (6)
C10	0.0224 (8)	0.0130 (7)	0.0207 (7)	0.0002 (5)	0.0011 (6)	0.0004 (6)
C11	0.0208 (8)	0.0107 (6)	0.0204 (7)	0.0018 (5)	0.0002 (6)	0.0028 (6)
C9	0.0199 (8)	0.0109 (7)	0.0208 (8)	−0.0001 (5)	0.0001 (6)	−0.0012 (5)
C12	0.0207 (8)	0.0123 (6)	0.0218 (8)	−0.0005 (6)	−0.0032 (6)	−0.0003 (6)
C16	0.0239 (8)	0.0130 (6)	0.0188 (7)	0.0006 (5)	−0.0007 (6)	−0.0008 (5)
C15	0.0200 (8)	0.0166 (7)	0.0231 (8)	−0.0019 (6)	−0.0027 (6)	−0.0002 (6)
C3	0.0227 (8)	0.0158 (7)	0.0268 (8)	−0.0027 (5)	0.0047 (7)	−0.0042 (6)
C2	0.0200 (8)	0.0162 (7)	0.0271 (8)	0.0010 (6)	−0.0022 (6)	−0.0041 (6)

### (E)-N-(4-Bromophenyl)-1-(quinolin-2-yl)methanimine Geometric parameters (Å, º)

**Table:**

Br1—C14	1.9021 (16)	C4—C3	1.374 (2)
N1—C6	1.372 (2)	C1—H1	0.9500
N1—C9	1.3264 (19)	C1—C6	1.419 (2)
N2—C10	1.282 (2)	C1—C2	1.370 (2)
N2—C11	1.411 (2)	C14—C15	1.392 (2)
C13—H13	0.9500	C10—H10	0.9500
C13—C14	1.387 (2)	C10—C9	1.474 (2)
C13—C12	1.387 (2)	C11—C12	1.399 (2)
C7—H7	0.9500	C11—C16	1.403 (2)
C7—C5	1.423 (2)	C12—H12	0.9500
C7—C8	1.363 (2)	C16—H16	0.9500
C5—C4	1.413 (2)	C16—C15	1.387 (2)
C5—C6	1.423 (2)	C15—H15	0.9500
C8—H8	0.9500	C3—H3	0.9500
C8—C9	1.423 (2)	C3—C2	1.417 (2)
C4—H4	0.9500	C2—H2	0.9500
			
C9—N1—C6	117.37 (13)	C15—C14—Br1	119.61 (12)
C10—N2—C11	118.23 (13)	N2—C10—H10	119.6
C14—C13—H13	120.5	N2—C10—C9	120.83 (14)
C14—C13—C12	118.95 (14)	C9—C10—H10	119.6
C12—C13—H13	120.5	C12—C11—N2	121.82 (14)
C5—C7—H7	120.2	C12—C11—C16	119.25 (15)
C8—C7—H7	120.2	C16—C11—N2	118.67 (14)
C8—C7—C5	119.59 (14)	N1—C9—C8	123.88 (14)
C7—C5—C6	117.34 (15)	N1—C9—C10	114.92 (13)
C4—C5—C7	123.26 (15)	C8—C9—C10	121.14 (13)
C4—C5—C6	119.37 (15)	C13—C12—C11	120.61 (14)
C7—C8—H8	120.5	C13—C12—H12	119.7
C7—C8—C9	118.90 (14)	C11—C12—H12	119.7
C9—C8—H8	120.5	C11—C16—H16	119.7
C5—C4—H4	119.8	C15—C16—C11	120.62 (14)
C3—C4—C5	120.37 (15)	C15—C16—H16	119.7
C3—C4—H4	119.8	C14—C15—H15	120.6
C6—C1—H1	119.8	C16—C15—C14	118.72 (14)
C2—C1—H1	119.8	C16—C15—H15	120.6
C2—C1—C6	120.33 (14)	C4—C3—H3	119.8
N1—C6—C5	122.81 (14)	C4—C3—C2	120.30 (16)
N1—C6—C1	118.13 (13)	C2—C3—H3	119.8
C1—C6—C5	119.05 (14)	C1—C2—C3	120.52 (15)
C13—C14—Br1	118.56 (12)	C1—C2—H2	119.7
C13—C14—C15	121.83 (15)	C3—C2—H2	119.7
			
Br1—C14—C15—C16	179.06 (11)	C6—N1—C9—C8	2.9 (2)
N2—C10—C9—N1	174.27 (14)	C6—N1—C9—C10	−174.18 (12)
N2—C10—C9—C8	−2.9 (2)	C6—C5—C4—C3	0.8 (2)
N2—C11—C12—C13	174.38 (13)	C6—C1—C2—C3	−0.4 (2)
N2—C11—C16—C15	−175.74 (13)	C14—C13—C12—C11	0.9 (2)
C13—C14—C15—C16	−0.3 (2)	C10—N2—C11—C12	46.8 (2)
C7—C5—C4—C3	178.98 (13)	C10—N2—C11—C16	−139.02 (14)
C7—C5—C6—N1	−2.1 (2)	C11—N2—C10—C9	−171.29 (13)
C7—C5—C6—C1	179.09 (13)	C11—C16—C15—C14	1.4 (2)
C7—C8—C9—N1	−2.5 (2)	C9—N1—C6—C5	−0.6 (2)
C7—C8—C9—C10	174.41 (13)	C9—N1—C6—C1	178.28 (13)
C5—C7—C8—C9	−0.3 (2)	C12—C13—C14—Br1	179.79 (11)
C5—C4—C3—C2	1.2 (2)	C12—C13—C14—C15	−0.8 (2)
C8—C7—C5—C4	−175.72 (14)	C12—C11—C16—C15	−1.4 (2)
C8—C7—C5—C6	2.5 (2)	C16—C11—C12—C13	0.3 (2)
C4—C5—C6—N1	176.16 (13)	C2—C1—C6—N1	−176.41 (13)
C4—C5—C6—C1	−2.7 (2)	C2—C1—C6—C5	2.5 (2)
C4—C3—C2—C1	−1.4 (2)		

### (E)-N-(4-Bromophenyl)-1-(quinolin-2-yl)methanimine Hydrogen-bond geometry (Å, º)

*Cg*1, *Cg*2 and *Cg*3 are the centroids of the N1/C5–C9, C1–C6 and C11–C16 rings, respectively.

**Table:**

*D*—H···*A*	*D*—H	H···*A*	*D*···*A*	*D*—H···*A*
C1—H1···Cg3^i^	0.95	2.67	3.3703 (16)	131
C4—H4···Cg2^ii^	0.95	2.79	3.4808 (17)	130
C7—H7···Cg1^ii^	0.95	2.85	3.5026 (16)	127
C13—H13···Cg3^iii^	0.95	2.69	3.4042 (16)	132
C16—H16···Cg2^iv^	0.95	2.70	3.3932 (15)	131

Symmetry codes: (i) −*x*+1, −*y*+1, −*z*+2; (ii) *x*, −*y*+1/2, *z*−3/2; (iii) *x*, −*y*+1/2, *z*−1/2; (iv) −*x*+1, −*y*+1, −*z*+1.
